# Draft genome sequence of *Azospirillum brasilense* JM 6A2 (DSM 1858)

**DOI:** 10.1128/mra.01505-25

**Published:** 2026-07-10

**Authors:** Kayla M. Clouse, Manuel Kleiner, Maggie R. Wagner

**Affiliations:** 1 Department of Plant Science, Pennsylvania State University 8082, University Park, Pennsylvania, USA; 2 Department of Ecology and Evolutionary Biology, University of Kansas 4202, Lawrence, Kansas, USA; 3 Kansas Biological Survey and Center for Ecological Research, University of Kansas 4202, Lawrence, Kansas, USA; 4 Department of Plant and Microbial Biology, North Carolina State University 6798, Raleigh, North Carolina, USA; Rochester Institute of Technology, Rochester, New York, USA

**Keywords:** plant-microbe interactions, plant growth-promoting bacteria, *Azospirillum brasilense*, nitrogen fixation, phytohormone synthesis

## Abstract

Here, we present the genome sequence of *Azospirillum brasilense* JM 6A2 (DSM 1858), which was isolated from maize roots in Ecuador. This genome provides a resource for studying the genetic basis of nitrogen fixation and phytohormone synthesis in this agriculturally important species.

## ANNOUNCEMENT

*Azospirillum brasilense* is a gram-negative, nitrogen-fixing bacterium that associates with the roots of various plant species (1). *A. brasilense* is of agronomic interest due to its ability to fix atmospheric nitrogen and synthesize phytohormones, which collectively enhance root development, facilitate nutrient acquisition, and can improve overall plant growth (2). Here, we present the genome sequence of *A. brasilense* JM 6A2, which was isolated from maize roots in Ecuador (3).

*A. brasilense* JM 6A2 (DSM 1858) was obtained from the Leibniz Institute DSMZ. The strain was grown on R2A media (4) for 48 h at 30°C, and then 50–100 mg of cells were collected. DNA extraction and sequencing were performed by SeqCoast Genomics (Portsmouth, NH, USA). DNA was extracted using the DNeasy 96 PowerSoil Pro QIAcube HT Kit (QIAGEN) after lysis in MagMAX Microbiome Bead Tubes (Thermo Fisher Scientific). Short-read libraries were prepared using the Illumina DNA Prep tagmentation kit and sequenced on an Illumina NextSeq 2000 (2 × 150 bp). Reads were demultiplexed and trimmed using DRAGEN v4.2.7 (5). Long-read libraries were prepared using the Native Barcoding Kit (Oxford Nanopore Technologies) and sequenced on a PromethION 2 Solo (R10.4.1 flow cell). Basecalling with barcode trimming was performed using Dorado v7.6.7 (super-accurate model; Oxford Nanopore Technologies). Illumina sequencing yielded 60× coverage (1,494,675 paired-end reads, mean length 148.6 bp), and Nanopore sequencing yielded 120× coverage (108,829 reads, mean length 8,252.2 bp).

The genome assembly was generated using Flye v2.9.6-b1802 with estimated genome size and plasmid detection (6). Long-read polishing was performed with Medaka v2.1.1 using the r941_min_sup_g507 model (7), followed by short-read polishing with Polypolish v0.6.1 using Illumina reads aligned with bwa v0.7.19 (8, 9). The final assembly comprised 7.46 Mb with a GC content of 68% and an N50 of 1.77 Mb. Assembly quality assessment using CheckM2 v1.1.0 (10) indicated 100% completeness and 0.66% contamination. Replicon assignments were determined using MOB-suite v3.1.0 (mob_recon) (11) and further supported by the presence of replication initiation and partition genes. Although pulsed-field gel electrophoresis (PFGE) was not performed, our data are consistent with PFGE results from previously sequenced conspecifics (12). The sizes and GC contents of the replicons were 3.06 Mb (68.5%) for the chromosome; 1.77 Mb (68.4%), 1.53 Mb (68.6%), and 941 kb (68.8%) for the chromids (AbJM6A2_p1, AbJM6A2_p2, and AbJM6A2_p3); and 168 kb (67.2%) for the plasmid (AbJM6A2_p4). The genome size, replicon number, and GC content of JM 6A2 are consistent with other sequenced *A. brasilense* strains, including the reference strain Sp7 (12). All contigs except AbJM6A2_p2 were classified as circular by Flye v2.9.6-b1802, indicating complete chromosomes, chromids, or plasmids. Genome annotation performed using Prokka v1.14.6 with the default bacterial database (13) predicted 6,689 coding sequences, 106 transfer RNAs, one transfer-messenger RNA, and 10 ribosomal RNA (rRNA) operons. Four complete rRNA operons are located on AbJM6A2_p2, three complete rRNA operons and one rRNA operon lacking the 5S subunit are located on AbJM6A2_p1, and two complete rRNA operons are located on the chromosome. Genes associated with plant growth-promoting traits were identified, including nitrogen fixation (nifH, nifD, and nifK) and indole-3-acetic acid biosynthesis (ipdC).

## Data Availability

The genome sequence of *A. brasilense* JM 6A2 (DSM 1858) is available in GenBank under accession number JBTNQK000000000. Raw Illumina and Oxford Nanopore Technologies sequencing reads are available in the Sequence Read Archive under accession SRR36429348 and SRR36429346, respectively.
